# Puerperal Insanity

**Published:** 1848-04-01

**Authors:** James Reid

**Affiliations:** PHYSICIAN TO THE GENERAL LYING-IN HOSPITAL, ETC.


### ON THE SYMPTOMS, CAUSES, AND TREATMENT OF PUERPERAL INSANITY.

BY JAMES REID, M.D.

PHYSICIAN TO THE GENERAL LYING-IN HOSPITAL, ETC.

(*Continued from our last Number.*)

THE first step towards the treatment of any disease, is the knowledge of its immediate and predisposing causes. Now, the opinion of the great majority of those who are in the habit of seeing puerperal mania is, as I before stated, "that it does not depend on the inflammation of the brain; but that its origin may be fairly traced to *cerebral irritation*, combined with great exhaustion of the nervous system generally." The analogy between *puerperal mania*, and that state of the system which exists in *delirium tremens*, holds good also in the treatment of the two diseases. The aim of the practitioner in either case is to subdue excitability without, at the same time, depressing further the vital powers; and as they are not simply diseases of mind, but dependent on functional derangement of the sensorium, the attention ought, of course, to be primarily directed to the physical treatment of that portion of the body, before attempting to remedy the mental mischief; or, in other words, we should endeavour to remove the cause, instead of combating the effect. The treatment will thus resolve itself into, 1. PHYSICAL—2. MORAL.

It is of importance to recollect that excitability is a frequent accompaniment of physical debility, and that violent paroxysms of mania may arise under these circumstances. To those unaccustomed to such a display of violence and apparent strength, the idea would, perhaps, immediately present itself, that depletion would be advisable in order to subdue them, and into this error many medical practitioners have formerly fallen. This leads us at once to the consideration of a remedy, which, even at the present day, occasionally finds advocates in such cases—viz., BLEEDING.

In PHRENITIS occurring after childbirth, there can be no doubt that very prompt treatment and active depletion are requisite, to afford a chance of recovery to the patient. Bleeding from the arm, leeches to the shaved head, blisters to the spine, repeated mercurial doses to prevent effusion, sinapisms to the feet, cold lotions, and all the other usual remedies in *acute inflammation,* are here most essential.

Experience, however, does not warrant such treatment in *puerperal mania,* notwithstanding the opinions of Puzos, Doublet, and some other authors. As many cases of puerperal fever (which after death have presented all the traces of peritoneal inflammation) arise after severe accidental hæmorrhage, or large bleedings from the arm, employed to arrest puerperal convulsions, (facts which I have frequently observed in hospital practice,) so does mania occasionally appear likewise after a copious loss of blood from any part of the system; and numerous cases might be adduced to prove this fact.

*Esquirol* narrates an instance of mania following accidental uterine hæmorrhage, which had lasted eight days. *Gooch* also mentions one which came on after an alarming hæmorrhage, and another which followed large bleedings from the arm. A very profuse leucorrheal discharge, in a case related by Haller, caused the patient to lose the faculty of thinking. The brain appears in these cases to be unable properly to perform its functions, owing to its not receiving its proper supply of blood.

It is mentioned by Esquirol, that such a prejudice formerly existed in favour of bleeding in cases of mania, that pregnant women were received into the Salpêtrière who had been previously bled, *as a precaution,* by practitioners, who knew that no bleeding would be practised in that hospital; and in one case, the patient had been bled thirteen times in forty-eight hours.

There is little doubt that, however violent the attack of mania may be, copious bleeding will be extremely prejudicial; and Pinel has clearly pointed out the liability of hurrying cases of simple insanity into a state of dementia by too energetic a treatment. Bleeding, too, will not, in many cases, *even temporarily* subdue the symptoms, but will cause them to become more inveterate and fixed, as the cerebral excitement is increased by the depletion. Gooch's cases give evidence as to the danger of great depletion, even when the pulse is quick, if it be *weak* at the same time. He says—"Bloodletting is not only seldom or never necessary, but, generally, almost always pernicious." (p. 162.) Dr. Gooch states, also, that he never met with a case requiring it.

From what experience I have had on this subject, I fully adhere to Dr. Gooch's opinion. I cannot recollect a case of *uncomplicated* puerperal mania in which the lancet was used; and in the most violent forms of the complaint, a few leeches to the head have been alone employed for the purpose of local depletion.

Cases have been narrated, both of this disease, and of delirium tremens, in which a small bleeding from the arm has been followed by speedy dissolution.

Dr. Rush has been accused by some of his medical compatriots of having caused much harm, by the directions, in his work on "Diseases of the Mind"—"to bleed copiously in maniacal excitement," and a writer in the "American Journal of Insanity" has lately stated—"that of 622 patients admitted into the asylum during the past year, *four* only were bled, and that in one case of the four alone did it prove at all beneficial."

EMETICS have been strongly recommended when the tongue is loaded, and the breath foul, at the commencement of the attack. A combination of ipecacuan with antimony appears to be the best form, when there is not great debility or anæmia.

PURGATIVES. Every obstetric practitioner of experience must be aware how frequently a whole train of alarming symptoms, occurring a few days after childbirth, and resembling the primary ones of puerperal fever, is at once subdued by an active aperient, or by a turpentine enema, which rids the patient of copious and vitiated dejections; the same good result has often resulted from their employment in puerperal mania also. Large evacuations of this description are, in fact, sometimes the first symptom of recovery in the patient. Even in cases of unusual exhaustion, constipation should, at least be avoided; and the bowels may be unloaded by means of gentle aperients and enemata of warm water. Many cases of general insanity have their origin in the state of the abdominal viscera *alone;* and in that dependent upon the puerperal state it often acts as a partial cause also.

Gooch's first case is very instructive, in showing to what an extent *hepatic* derangement may be concerned in causing puerperal mania, and the good effects of purgatives in removing it. The lady "had suffered in *two* confinements from this malady, each attack being *preceded by jaundice.* She was also completely jaundiced before her third confinement, but it was *on this occasion* removed by purgatives previously to the occurrence of labour, and she this time escaped mental derangement." —p. 112.

The *form* of aperient will, of course, vary according to the nature of the case, and the condition of the patient. I have found ʒj. of the pulvis jalapæ compositus, given in treacle as an electuary, answer the purpose very well in several cases, and this may be repeated at intervals if required. Dark fœtid evacuations are often dislodged; and many instances might be cited, in which great improvement was immediately a consequence. Should there be a wish to get rid of the secretion of milk as soon as possible, the hydragogue aperients will be best adapted for the purpose.

ANODYNES.—Almost all authors on this subject, whether the mania be general or puerperal, recommend the employment of this class of medicines, taking the precaution previously of properly evacuating the bowels. As in delirium tremens, opium, in its different preparations, may be given in larger doses than under ordinary circumstances. It has been considered, however, as a doubtful remedy by Burns, and an objection has been raised that it increases cerebral congestion. The complaint, however, is one of *irritation* generally, more than *congestion,* and opium, in most cases, will allay this. Opiates seem peculiarly adapted to puerperal cases, especially when combined with some diffusible stimulus, such as ammonia, and more especially with camphor. *Small*

doses of opium will, in many cases, increase irritability, instead of allaying it; and it is a better plan, in general, to administer a large dose at night, and the effect may afterwards be kept up by repeated but smaller doses.

The acetate or muriate of morphia, in quarter-grain doses, may be given at intervals; but I have frequently known half a grain, and even one grain, given at short intervals in otherwise intractable cases, with good effect; and this has been increased by combining with the morphia half-grain doses of the antimonii pot. tartras.

Dover's powder is another form of similar combination, which often proves a valuable remedy.

An occasional change in the anodyne is advisable in those cases which require the daily exhibition of such a remedy. Thus, half a grain of muriate or acetate of morphia may be administered at one time, a drachm of tinct. hyoscyami at another, and ten grains of Dover's powder on a third occasion; thus varying the form, when the repetition of the same medicine seems to diminish its effect.

There are some instances in which opium, in any shape, gives no relief in procuring sleep, but, on the contrary, appears to aggravate the insomnia and irritability.* In one such case, I found the employment of the hydrocyanic acid attended with the most beneficial effects. Five-drop doses of the diluted acid, in camphor julep, at intervals of four hours, were administered to the lady, and gradually procured a calm state of mind, and some refreshing repose.

The cannabis Indicus, or Indian hemp, has been known frequently to succeed in procuring rest, after the different preparations of opium had failed; the tincture is the best form, and is employed in doses of from twenty to sixty drops. It has, I understand, been used in the Hanwell Asylum with much benefit.

As it is a great object to break the *continuance* of this sleeplessness, in such cases the occasional use of the *chloroform* vapour will be found valuable. I have had an opportunity of seeing more than one case in which it not only induced sleep, which had previously been absent for four or five nights and days, but the patient on recovering from its effects, was found to be quite tractable and free from violence.

The inhalation of ether had been tried by M. Cazenave, of Paris, in the case of a lunatic female who had rested neither night nor day for five months, and in which it induced tranquillity. (Med. Gazette.) M. Jobert, in a similar case, exhibited it with the good effect of inducing sleep, and restoring, temporarily, a state of rationality. (Brit. and For. Review.) M. Bouvier tried ether, also, in a case of puerperal mania, with very beneficial results. In this case there had been no sleep for a fortnight before using the ether; its use was followed on two occasions by "*un calme de quelques heures.*" (Bull. de l'Academie; Brit. and For. Med. Review.) I am bound, however, to add, that in some cases in which it had been tried by other practitioners, no beneficial effect was produced.

As a sedative application, the employment of the *warm* or *tepid bath*

* In a case which I am at present attending, with Mr. Thistleton Dyer, the patient, who was attacked by puerperal mania on the eighth day after confinement with her first child, occasionally passes sixty hours without sleep.

has been found of great service in cases of puerperal mania; it allays the general irritability, causes the skin to perform its functions more healthily, tends to restore the secretions to a proper state, and soothes the patient. Iced lotions to the heated scalp may be applied at the same time. Many authors speak most highly of the effects produced on females by the use of such baths, especially when any suppression has occurred.

In some cases, the cold bath, the shower bath, and the practice recommended by Dr. Currie—viz., placing the patient in an empty bath, and pouring water on the head, have been attended with marked benefit. In all these forms it is better, however, to commence with the water tepid, and *gradually* to lessen the temperature in the succeeding applications.

Numerous instances exist, in which the tonic effect of the *shower* bath has produced excellent results, but it has been employed at a period of some weeks after parturition.

When the patient exhibits great watchfulness and inability to sleep, notwithstanding the employment of all sedatives, and this is combined with unusual irritability of manner and quick pulse, the case requires our most anxious attention, and every method possible to allay such excitement should be in succession tried. The room should be darkened, and kept perfectly quiet and cool; the covering on the bed should not be more than is sufficient; a mattress should be substituted for the feather bed, if the latter be used; and it is most essential that a nurse endowed with good sense and experience should be in attendance.

COUNTER-IRRITATION is sometimes of considerable advantage under such circumstances, and a blister to the spine, or dry-cupping over that part, will sometimes produce excellent effect. *Esquirol* speaks very favourably of blisters in the later stages of this form of insanity, when applied between the shoulders.

In the *adynamic* form, attendant upon *undue lactation*, it is *especially* requisite to avoid any depletion, or low diet. *Sedatives* are as important as in the other cases, and in addition to these, the use of tonics, such as quinine, bitter infusions, with the mineral acids, the various preparations of iron, the moderate use of wine and beer, and, if possible, after a time, a change to the invigorating breezes of the sea-side, or a quiet village, will be advisable.

One of the best means of lessening the irritability of the brain and the want of sleep, is shaving the head, and a persevering employment of refrigerant lotions to that part.

MORAL TREATMENT is of necessity required in the management of all cases of insanity, but by no means supersedes the physical. In the peculiar form we are now considering, it is especially requisite to attend most carefully, *first*, to the healthy state of the bodily secretions, as without this precaution, many cases have withstood all the moral remedies employed *for years*, and yet at the end of this period, by judicious means applied to the general state of health, they have yielded in a few weeks, and have been cured.

The suppression of natural evacuations is peculiarly liable to form one of the features of puerperal mania, but after these have been restored to their healthy and usual function, *then* the skilful management of the patient's mind becomes essentially necessary.

The moral management is simply that which is required in other forms of insanity.

The first point of importance is to keep the patient in as complete a state of QUIETUDE as possible. Every source of irritation, whether by noise, light, &c., should be speedily got rid of, so as to aid the intention of procuring rest for the excited cerebral organs. The presence of intimate friends and relations at this time is so far from being a comfort or advantage to the patient, that it is considered by every practitioner of experience to be absolutely prejudicial, unless under peculiar circumstances. If the patient still retain her love for them, there is a constant source of excitement in seeing or talking with them; whilst, on the other hand, should aversion assume the place of strong affection towards them, as is very frequently the case, it is still more requisite to avoid these meetings.

The withdrawal of the mind from former scenes and associations, and giving a change to the direction of the thoughts and recollections, is one of the principal aims to be attained; and the presence of strangers is better adapted to this end, as the chain of ideas is more likely to be interrupted and broken. With the same intention, of disturbing the association of ideas, change of scene is desirable after a certain time, so that the mind should not dwell upon the same objects which were presented to it by the locality in which the aberration of mind first occurred.

It is frequently noticed, also, that insane patients are able to exert more self-control before strangers than when surrounded by their relatives.*

The infant should be removed from the mother, in the generality of cases, as it is a great source of irritation by its cries, and precaution is otherwise constantly required, least by some sudden aversion to it, its life may be attempted by her. Such a case occurred not long since, in one of the lying-in hospitals I have mentioned, in which the infant was destroyed. In many cases there is such indifference towards the child, and to all other relations, that their absence is no punishment to the patient. The medical attendant will be the best judge as to when this temporary seclusion may terminate; as, after a time, a break in the monotony of it may prove beneficial, but great prudence is always required in trying the experiment.

It becomes, under these circumstances, of the greatest importance that the *attendants* should be *peculiarly adapted* for their duty; the usual domestics, and even the experienced monthly nurse, are not so valuable as one who is accustomed to the care of this class of patients, and it is advisable, therefore, to replace them. A vigilant, firm, though kind superintendence, soothing violence, encouraging and cheering despondency, soon produces its effect; and a nurse who is skilful and experienced in these cases, will have much more moral control than

* Complete isolation from friends appears to be one of the most important items in the treatment of all severe cases of insanity. It was remarked by Dr. Willis formerly, that foreigners who were brought from the continent to be placed under his care were much sooner cured than English patients; and Esquirol has observed the same fact in relation to the more speedy cure of provincial patients, as compared with the inhabitants of Paris.

others who are unaccustomed to the varying symptoms of the complaint, and will in many instances thus avoid the necessity of bodily restraint.

The control is not the less effectual for being mild, and those accustomed to the office may evince much quiet determination, without any imperious, authoritative manner or irritating behaviour. There is here no needless thwarting every harmless wish of the patient, which increases violence and begets hatred; for even maniacal patients are often sufficiently acute in detecting injustice or oppression. Dr. Conolly mentions two female cases received into Hanwell, in 1845, who had both become insane in the puerperal state. One was an irritable patient, easily excited; the other, a delicate and timid woman, easily alarmed. Both had, previously to their admission, been subjected to *severe restraint*, and began to recover almost as soon as admitted, under kinder treatment. One remained three months in the asylum, and the other only *one* month. No arguments should be entered into, in order to convince such patients that they are labouring under delusion; they prove of no avail, but rather confirm them in their opinions, as they are perfectly contented and convinced by their own chain of reasoning, although resting on false data, and often absurd.

Judicious and well-timed conversation often relieves the mind of its terrors or doubts, and experience has amply proved that the comforts of religion are fully realized by many insane patients. In the Glasgow Lunatic Asylum Reports it is mentioned that a religious conversation, and well-timed application of a scriptural quotation, had the full effect of putting an end to a previously determined resolve to commit suicide.

A quotation from Sir W. Ellis's work should always be borne in mind as to this latter tendency—"There is no form of insanity in which attempts at self-destruction are more unexpectedly and suddenly made, than in the puerperal form." The patient should never be alone for an instant, not even when apparently asleep; for, connected with this *suicidal tendency*, great cunning and ability are displayed in the attempts to avoid the notice of the attendants: many instances of regular and progressive preparations for eluding suspicion and accomplishing their purpose are upon record. Every dangerous article should, under such cases, be removed, even to the pocket handkerchief and garters, the windows should be unostentatiously guarded, and a quiet, though active surveillance constantly kept upon every action of the patient.

A question has often been raised, whether patients labouring under puerperal insanity should be removed to an asylum or not; but the general opinion is against it—*i.e.*, in private practice, unless the case should subside into a chronic and lingering form. If change of scene be deemed requisite, it is better that the patient should be removed at first to a quiet country village, or to the sea-side, under the care of an experienced nurse, but the frequent visits of the medical attendant will here be advisable, on many accounts; the best nurses should not have too much power confided to them in these cases, and the uncertain visit of the practitioner is a safeguard against neglect or injudicious measures. The friends of the patient should also pay occasional visits, to examine into the domestic arrangements and comforts of the place, without, however, seeing the patient herself, or, at least, refraining from doing so until the medical adviser sees the propriety of trying the effect of their presence upon her. One injudicious and too premature a visit has, in many cases, retarded the cure for weeks, and even months, by recalling distressing associations to the mind.

One very important item in the treatment of these cases, is great attention to the *dietary* of the patient.

In the great majority of cases, it is requisite to give nutritious though light food at an early period of the complaint, as it must be remembered, that want of an adequate supply has in many cases of insanity been alone sufficient to induce the disease. Strong broths, or beef-tea, farinaceous articles of diet, milk, eggs, &c., may be allowed, the bowels at the same time being carefully attended to; and as the tongue and pulse improve, a well-watched increase of nutriment will be required. In lunatic asylums, the diet-table has much improved within the last few years with great advantage, and in this peculiar form of it, especially when arising from *undue lactation*, a generous diet is advisable at once, combined with tonics and chalybeates. We must not, in such cases, wait until the patient asks for food, but it must be rather pressed upon her at regular intervals; in fact, it is of high importance that great regularity should be observed as to the time of meals, as well as to the hour of retiring to rest, and rising in the morning.

When puerperal insanity has once occurred, it will be an imperative duty on the part of the medical attendant, at any future pregnancy, to insist strongly on the necessity of quiet, and the absence of all unnecessary excitement, by carefully watching symptoms which bear the appearance of premonitory ones, and taking measures to prevent their continuance; the cerebral irritation may be sometimes checked *in limine*, and the case conducted to a favourable termination, without any further bad effects arising from it.

The plan of treatment to be adopted in those cases of insanity which occur *during pregnancy* is not materially different from that which is applicable after parturition, or weaning. Anxiety must, however, always exist in the minds of the practitioner and friends of the patient, until parturition and its effects have passed over.

Occasionally instances have been met with in which the symptoms have become so urgent, that it was deemed necessary to terminate the period of gestation by artificial means before the proper time.

A case is mentioned in the "American Journal of Medical Science," of a lady in whom it was found requisite to induce premature labour on *three* different occasions, for aberration of mind during gestation.

When insanity during the pregnant state is complicated with a tendency to epileptic convulsions, our treatment in some cases appears totally inefficacious in warding off a serious result.

I was requested, on the 29th of January last, to meet Mr. Hugman, of Great Ormond-street, in consultation on an unfortunate case of this description. The patient, a lady, aged thirty-eight, had borne seven children, and was at this time advanced to the middle of the last month of pregnancy. She was a healthy-looking woman, of florid countenance, naturally of an excitable temperament, but had never previously evinced any symptoms of this complaint. She had always recovered speedily from the effects of labour, and had suckled several of her infants. For the last twenty years, she had been subject to slight fits at each period of menstruation, but never except at those times. Her husband thinks they were of an hysterical character, but from the description of a near relative, there can be no doubt that they were of an epileptic nature. Latterly, too, they had become more decided, and of longer duration. On first seeing the patient, she was tranquil, but had an expression of anxiety, the pulse was rather quick, but soft, the tongue clean, *no pain in the head,* bowels inclined to constipation, but easily acted on by medicine. She had had little or no sleep for the last two nights, and not much during the past week. She had suddenly awakened her husband three nights since, to inform him that it had been notified to her by the Bible, that she should die in her approaching confinement, and that she was an instrument chosen of God for some great purpose. This, she repeated on our visit, in a mysterious manner, but could give no further explanation, her manner being placid, but accompanied by a peculiar expression of the eyes. As the want of sleep appeared to be the principal symptom requiring attention, we decided upon giving half a grain of acetate of morphia at bed-time, and a gentle aperient in the morning; the diet to be light and nutritious.

At our next meeting, on the 30th, we found the patient quite calm; she had enjoyed much refreshing sleep during the night; the pulse was soft and natural; there was no pain in the head whatever, and she did not recollect the delusions of the previous day. She said that she felt perfectly comfortable in every respect, except that she was troubled by slight recurrent pains in the abdomen, which it was considered might be caused either by the aperient, or by the commencement of labour. Mr. Hugman found no evidence, however, of the latter fact on examining the os-uteri.

The patient was now apparently in so improved and favourable a condition, that it was thought unnecessary that I should again see her, unless any change for the worse should occur; but it was understood that she was never to be left alone, and that great additional care was to be taken of her during, and after her labour.

During the night, however, decided epileptic paroxysms attacked the patient, and in the afternoon of the next day recurred with great violence and frequency. Blood was abstracted from the arm by Mr. Hugman, leeches applied to the temples, cold lotions to the head, hot bottles to the feet, mustard cataplasms to the calves of the legs, but without any advantage.

Owing to my being engaged elsewhere, I was unable to meet Mr. Hugman until eleven, P.M., but the patient was at that time moribund, having been unconscious for several hours, the pupil not acted on by strong light, and the convulsive paroxysms recurring with violence every few minutes, until death took place at half-past one, A.M., without any further signs of labour being observed.

The following is a case which occurred in the General Lying-in Hospital, for the notes of which I am indebted to Mr. Smith, the house-surgeon at that period :—

H. C., aged 22, of dark complexion, with black hair, was delivered of her first child on the 14th of March, 1847. She had enjoyed good health previously to her admission, and was progressing favourably up to the eighth day from her confinement. In the afternoon of this day, she had been visited by some of her friends, and in the evening was observed to be crying, and in a very nervous, irritable state, complaining that some persons had been telling falsehoods about her at home. She could not be pacified, but, on the contrary, became much more excited on attempting to soothe her. A draught containing opium, ammonia, and camphor was given at night, and a purgative in the morning. She continued in the same state of excitement, however, and in a cold, clammy perspiration, the pulse quick and hard, bowels costive; she had had no sleep during the night, and refused to take food. Twenty-five drops of liquor opii sedativus were given every four hours, and continued for two days without the least benefit; her symptoms became more aggravated; she was quite unconscious of having an infant, and her milk gradually diminished from the commencement of the attack. She was at times exceedingly violent, making use of obscene language, and screaming. The head was shaved, the ice-cap applied to it, leeches to the temples, and a blister to the spine; aperients being occasionally administered also. Calomel and opium were now given every four hours, and continued for three or four days without the gums becoming effected, or the bowels acting, although enemata were also occasionally employed. No pain or tenderness of the abdomen was present, but it was found necessary to evacuate the bladder occasionally by means of the catheter. The temperature of the body was low, the tongue was white and furred, the countenance wild, eyes large and rolling, and head at times hot. She could talk for a time very rationally; but owing to her sudden fits of violence, it was found unsafe to trust her alone for a moment. As no improvement was taking place, it was thought expedient to remove her to an asylum, on April 8th, from which she was discharged cured, in about four months.

Mrs. ——, aged 20, had been distressed by some family occurrences, which caused her much anxiety Four days after her first confinement, she became much excited, and at length exceedingly violent, swearing and using most obscene language, although at other times a lady of most correct demeanour. As the usual treatment seemed to produce no good effect, and she had taken an inveterate dislike to her husband and child, it was thought advisable to remove her to a cottage in the country, under the charge of two experienced nurses. In about five months, she was restored to perfect health, and on her asking to see her husband, he was immediately allowed to visit her. She has since this period borne several children, but although of a nervous temperament, has had no return of the complaint.

Mrs. ——, aged 30, of a timid, sensitive disposition, was confined of her first child on December 25th, and was going on exceedingly well, until six weeks after, when she suddenly expressed a wish to bid adieu to her friends, as she thought herself dying. A great aversion to her husband, child, and to a near relative who was staying with her, soon after followed, and she refused to allow the infant to be applied to the breasts. Violent mania supervened, and she attempted suicide; but her language was not at all indecorous, and she sang with great taste almost constantly. The head was shaved, the ice-cap applied to it, a few leeches to the temples, and blisters to the spine; opium was given, followed by gentle purgatives, but no decided improvement took place for › time. She became exceedingly weak, and her nourishment was therefore given in a concentrated form, and frequently. At length, evident signs of amendment were observed; her mind gradually recovered its tone, as her general health improved, and she was quite well within six months.

---

